# An Ultra-Robust, Highly Compressible Silk/Silver Nanowire Sponge-Based Wearable Pressure Sensor for Health Monitoring

**DOI:** 10.3390/bios15080498

**Published:** 2025-08-01

**Authors:** Zijie Li, Ning Yu, Martin C. Hartel, Reihaneh Haghniaz, Sam Emaminejad, Yangzhi Zhu

**Affiliations:** 1 Terasaki Institute for Biomedical Innovation, Los Angeles, CA 91367, USA; zijie.li@lglab.ac.cn (Z.L.); mhartel@g.ucla.edu (M.C.H.); rhaghniaz@terasaki.org (R.H.); 2 Department of Materials Engineering, University of Southern California, Los Angeles, CA 90089, USA; 3 Department of Chemical Engineering, Stanford University, Stanford, CA 94305, USA; ningyu23@stanford.edu; 4 Department of Bioengineering, University of California Los Angeles, Los Angeles, CA 90095, USA; 5 Department of Electrical and Computer Engineering, University of California Los Angeles, Los Angeles, CA 90095, USA

**Keywords:** pressure sensors, silk fibroin, silver nanowire, wearables

## Abstract

Wearable pressure sensors have emerged as vital tools in personalized monitoring, promising transformative advances in patient care and diagnostics. Nevertheless, conventional devices frequently suffer from limited sensitivity, inadequate flexibility, and concerns regarding biocompatibility. Herein, we introduce silk fibroin, a naturally occurring protein extracted from silkworm cocoons, as a promising material platform for next-generation wearable sensors. Owing to its remarkable biocompatibility, mechanical robustness, and structural tunability, silk fibroin serves as an ideal substrate for constructing capacitive pressure sensors tailored to medical applications. We engineered silk-derived capacitive architecture and evaluated its performance in real-time human motion and physiological signal detection. The resulting sensor exhibits a high sensitivity of 18.68 kPa^−1^ over a broad operational range of 0 to 2.4 kPa, enabling accurate tracking of subtle pressures associated with pulse, respiration, and joint articulation. Under extreme loading conditions, our silk fibroin sensor demonstrated superior stability and accuracy compared to a commercial resistive counterpart (FlexiForce™ A401). These findings establish silk fibroin as a versatile, practical candidate for wearable pressure sensing and pave the way for advanced biocompatible devices in healthcare monitoring.

## 1. Introduction

Wearable pressure sensors have become indispensable tools in modern healthcare, enabling enhanced patient monitoring, diagnosis, and treatment [1,2,3,4]. From pressure-sensitive bandages in wound management to intraoperative and postoperative pressure tracking, these pressure sensors offer critical insights in diverse clinical settings [5,6,7,8]. As healthcare adapts to rapid technological advances, wearable pressure sensors are increasingly vital for delivering accurate, real-time data that support more effective and personalized patient care.

Despite recent advances, existing wearable pressure sensors continue to face significant hurdles related to sensitivity, durability, and biocompatibility. Achieving higher accuracy and truly continuous monitoring, especially of vital signs, remains a pressing challenge [9]. Moreover, long-term skin contact can provoke discomfort or allergic reactions when materials do not meet stringent biocompatibility standards [10,11]. In an era where patient comfort and safety are paramount, addressing these limitations is essential for widespread clinical adoption.

Silk fibroin, a natural, protein-based polymer produced by silkworms, has emerged as a promising solution to these challenges [12,13,14,15]. Its inherent biocompatibility ensures that prolonged skin contact does not elicit irritation or hypersensitivity, while its robust mechanical properties (high tensile strength, elasticity, and toughness) enable sensors to maintain sensitivity and functionality under repeated deformation [16,17,18]. These features position silk fibroin as an ideal substrate for fabricating next-generation pressure sensors that combine accuracy, comfort, and long-term stability.

Silk-derived pressure sensors hold tremendous potential across a range of medical applications [19]. In wound care, for example, a silk-based pressure-sensitive bandage could detect subtle changes in pressure distribution, signaling early complications and guiding timely interventions to improve healing outcomes [20,21]. In neonatal monitoring, integrating ultra-soft, silk-based sensors into garments or footwear could provide the continuous, noninvasive tracking of a baby’s movements, heart rate, and respiration without compromising comfort [22]. Similarly, in rehabilitation or sports medicine, silk-based sensors embedded in wearable garments could deliver precise pressure mapping and motion analysis, facilitating better injury prevention and performance optimization [23,24].

Here, we introduce a silk fibroin-based wearable pressure sensor (SWPS) that converts a wide spectrum of pressure changes into reliable electrical signals. Harnessing silk fibroin’s exceptional biocompatibility, the SWPS conforms comfortably to the skin for continuous physiological monitoring without provoking irritation. By uniting high sensitivity, mechanical flexibility, and user comfort, with multiple sensing functions embedded in a single, low-profile patch [25,26,27], this platform unlocks new possibilities in medical technology, from more precise diagnostics to real-time patient monitoring that can ultimately enhance clinical outcomes.

## 2. Experimental Sections

### 2.1. Materials

Sodium carbonate (anhydrous, Thermo Fisher Scientific, Waltham, MA, USA), lithium bromide (Sigma-Aldrich, St. Louis, MO, USA), and methanol (Thermo Fisher Scientific, USA) were used as received. Ethylene glycol (Sigma-Aldrich, USA), polyvinylpyrrolidone (PVP; Sigma-Aldrich, USA), sodium chloride (Sigma-Aldrich, USA), and silver nitrate (AgNO_3_; Sigma-Aldrich, USA) were employed without further purification. Dulbecco’s phosphate-buffered saline (DPBS; Gibco, Thermo Fisher Scientific, USA) served as the aqueous buffer for all in vitro assays. Silkworm cocoons (Bombyx mori) and copper tape (for electrode connections) were obtained via Amazon (Seattle, WA, USA). Human dermal fibroblasts (HDF; ATCC, Manassas, VA, USA) were cultured in Dulbecco’s Modified Eagle Medium (DMEM) supplemented with fetal bovine serum (FBS; Gibco, Thermo Fisher Scientific, USA) and 1% penicillin–streptomycin (100 U mL^−1^ penicillin and 100 µg mL^−1^ streptomycin; Gibco, Thermo Fisher Scientific, USA). Cell viability assays were performed using PrestoBlue™ Cell Viability Reagent (Invitrogen, Thermo Fisher Scientific, Carlsbad, CA, USA). The HDF cell was sourced from American Type Culture Collection (ATCC, Manassas, VA, USA)

### 2.2. Silk Sponge Fabrication

Silkworm cocoons were first degummed to remove sericin. Cocoons were cut into small pieces and boiled in an aqueous sodium carbonate solution (cocoon: Na_2_CO_3_ mass ratio = 1:0.85) for 30 min. The resulting fibroin fibers were thoroughly rinsed in deionized (DI) water and then soaked in fresh DI water for 90 min, replacing the water every 30 min to ensure the complete removal of residual sericin. After soaking, the degummed silk was dried in an oven at 37 °C for 24 h. Dried silk fibers were dissolved in a 9.3 M lithium bromide solution at a silk:LiBr mass ratio of 1:3.23. The mixture was incubated until fully solubilized, then dialyzed against DI water for 72 h using a 3500 Da molecular weight cutoff membrane, with at least three water changes per day. The purified silk fibroin solution was centrifuged at 8000× g rpm and 4 °C for 20 min to remove particulates. The supernatant was passed through a sterile mesh filter and aliquoted for storage at −80 °C. The concentration of regenerated silk fibroin was determined by freeze-drying a known volume of solution and weighing the resulting solid. For sponge fabrication, aliquots of silk fibroin solution were thawed and diluted with Milli-Q water to final concentrations of 1, 2, 3, 4, 5, and 6% (*v*/*v*). Each solution (volume sufficient to fill a 48-well plate; well diameter = 10 mm) was dispensed into individual wells. The plates were frozen at −80 °C and lyophilized for 48 h to yield porous silk sponges. To induce β-sheet crosslinking [28,29], the dry sponges were immersed in pure methanol for 2 h, then washed extensively with Milli-Q water to remove residual solvent. Finally, the sponges were freeze-dried for an additional 48 h and stored in a desiccator until further use.

### 2.3. Preparation of AgNW

Silver nanowires (AgNWs) were synthesized via a modified polyol method. In a typical preparation, 20 mL of ethylene glycol containing polyvinylpyrrolidone (PVP, 1 g) and sodium chloride (NaCl, 14 mg) was stirred at room temperature for 10 min, then heated to 160 °C and held at that temperature for 20 min. A separate 0.14 M silver nitrate (AgNO_3_) solution (10 mL) was then introduced into the hot PVP/NaCl solution via syringe pump at a flow rate of 10 mL h^−1^ while maintaining the reaction temperature at 160 °C. After complete addition, the mixture was further heated at 160 °C for an additional 30 min, during which the solution turned gray–green, indicating AgNW formation. The resulting suspension was cooled to room temperature and washed twice by centrifugation (6000× *g* rpm, 10 min) with acetone and then with Milli-Q water to remove residual PVP and salts. Finally, the purified AgNWs were redispersed in ethanol and concentrated to approximately 40 mg mL^−1^ for subsequent electrode fabrication.

### 2.4. SWPS Fabrication and Assembly

Silver nanowire (AgNW) coatings were prepared based on our previously established protocol [30]. AgNWs were first dispersed in ethanol and sonicated for 10 min. The resulting suspension was then vacuum filtered through a 100 nm pore-size porous membrane, yielding a uniform conductive film of AgNWs on the membrane surface. To transfer this film onto the silk sponge, the AgNW-coated membrane was placed in intimate contact with one face of the sponge, and gentle manual pressure was applied to affect the transfer of nanowires. After deposition on the first face, the procedure was repeated on the opposite face of the sponge to obtain a bilayer AgNW electrode coating, thereby completing sensor assembly.

### 2.5. Cytocompatibility Evaluation

Human dermal fibroblasts (HDFs; ATCC) were maintained in Dulbecco’s Modified Eagle Medium (DMEM) supplemented with 10% fetal bovine serum (FBS) and 1% penicillin–streptomycin at 37 °C in a humidified 5% CO_2_ atmosphere. Cells were passaged upon reaching ~90% confluency and subsequently seeded at a density of 1 × 10^4^ cells per well onto sterilized silk sponge or fully assembled SWPS devices housed within 24 well plates. Cytotoxicity was evaluated on days 1 and 5 using the PrestoBlue™ Cell Viability Assay (Invitrogen, Carlsbad, CA, USA). Briefly, PrestoBlue reagent was diluted 1:9 (*v*/*v*) in a complete culture medium, and 1.5 mL of this mixture was added to each well. Samples were incubated at 37 °C in the dark for 1.5 h, after which fluorescence was measured (excitation 530 nm, emission 590 nm) using a microplate reader. On day 5, a live/dead staining assay was performed to assess cell viability and morphology. Silk substrates were rinsed twice with Dulbecco’s phosphate-buffered saline (DPBS), and a staining solution containing ethidium homodimer-1 (20 µL) and calcein AM (5 µL) in 10 mL DPBS was prepared. Each sample received 500 µL of staining solution and was incubated at 37 °C in the dark for 15 min. Fluorescence images were then captured using a Keyence BZ-X700 series microscope (Keyence, Osaka, Japan), with live cells appearing green (calcein AM) and nonviable cells appearing red (ethidium homodimer-1).

### 2.6. On-Body Physiological Performance Evaluation

The on-body evaluation was conducted on healthy adult individuals without heart conditions, diabetes, or chronic pain, adhering strictly to the protocol sanctioned by the Institutional Review Board at the University of California, Los Angeles (IRB#17-000170). For on-body evaluation, SWPS devices were affixed to various anatomical locations (e.g., wrist, carotid artery, chest, and joints) using conductive copper tape at one end. The free face of each sensor conformed directly to the skin surface, such that any externally induced deformation (e.g., arterial pulse, muscle contraction, and joint flexion) compressed the porous silk–AgNW sponge and produced a measurable change in capacitance. In this configuration, physiological movements and pressures applied to the exposed side of the SWPS were transduced into real-time electrical signals, enabling the precise mapping of diverse body-generated forces.

### 2.7. Vehicular Tire Compression Endurance Test

SWPS devices were affixed to the left front tire of a passenger vehicle using conductive copper tape, with the free end of each sensor connected to an LCR meter (measuring capacitance, inductance, and resistance) for a continuous readout. The vehicle was driven at a constant speed of 5 mph (≈8 km/h) over a flat roadway segment for a total duration of 200 s, during which time capacitance signals were recorded in real time. Immediately following this test, a commercial resistive pressure sensor (FlexiForce™ A401, FlexiForce, Barneveld, The Netherlands) was installed in the same location and secured under identical conditions. The vehicle was then driven for an additional 200 s at 5 mph, and resistance changes from the commercial sensor were logged continuously for direct comparison. Continuous signal acquisition for both sensors was conducted at a sampling rate sufficient to resolve individual tire crossings (≥10 Hz).

## 3. Results and Discussion

### 3.1. Device Design of the SWPS

**Figure 1:**
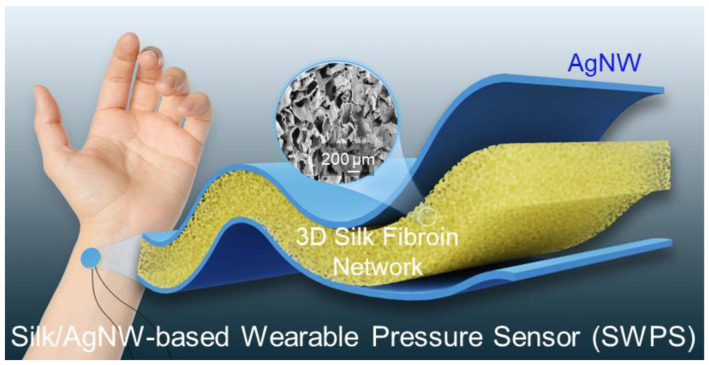
Schematic illustration of the Silk/AgNW wearable pressure sensor (SWPS) design. A 3D porous silk fibroin sponge (yellow) is sandwiched between flexible silver nanowire (AgNW) electrodes (blue) to form a highly conformal capacitive sensor. When affixed to the wrist, the silk network compresses under applied pressure, enabling real-time, biocompatible monitoring of physiological signals. The inset shows an SEM image of the porous silk fibroin microstructure, highlighting its interconnected network.

Silk fibroin-based pressure sensors have excellent biocompatibility and can be placed directly on the surface of the human body to capture pressure feedback from various bodily regions. Figure 1 shows an overview of an SWPS worn on the human wrist. The sensor’s 3D porous structure imparts it with exceptional breathability. This feature facilitates long-term measurements without causing discomfort or hindrance to the wearer. In scenarios that require long-term use, such as continuous patient monitoring or prolonged physical activity, the breathability of the sensor is very important. Both sides of the silk sponge were coated with silver nanowires (AgNWs) to create the electrodes which serve as the pressure transducer. When pressure is applied to the sensor, the middle porous structure of the silk sponge compresses, leading to changes in its capacitance value. This distinctive feature enables our SWPS to provide precise measurements of repetitive and extended pressure fluctuations.

### 3.2. Benchtop Evaluation of the SWPS

**Figure 2:**
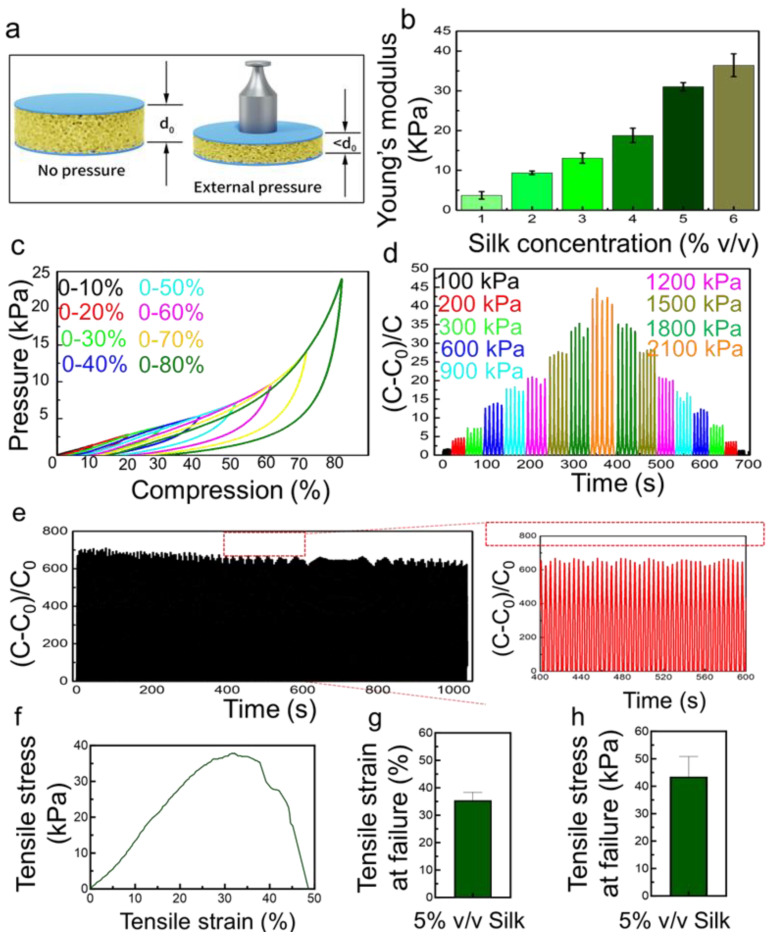
Characterization, optimization and stability evaluation of SWPS. (**a**) Schematic of the capacitive pressure measurement: in the unloaded state, the porous silk sponge has an initial thickness *d*_0_, and under external pressure, the sponge compresses to a thickness < *d*_0_, altering the capacitance. (**b**) Measured Young’s modulus of SWPS sponges as a function of silk fibroin concentration (1–6% *v*/*v*), showing a monotonic increase from ~4 kPa (1%) to ~35 kPa (6%). (**c**) Compression–pressure profiles at increasing strain levels, demonstrating that higher compressive deformations require larger pressures while maintaining reproducible rebound behavior (minimal hysteresis) across repeated loading. (**d**) Capacitance change (ΔC) versus applied pressure for a 5% *v*/*v* SWPS under sequential loads of 200, 300, 600, 900, 1200, 1500, 1800, and 2100 Pa; the sensor exhibits a linear response (R^2^ = 0.99) with high sensitivity (0.0187 Pa^−1^). (**e**) Long-term cycling stability over 1000 s (14 cycles min^−1^) under repeated loading/unloading, with the inset highlighting consistent amplitude and negligible signal drift throughout the endurance test. (**f**) Representative stress–strain curve of 5% *v/v* SWPS under uniaxial tension. (**g**) Ultimate tensile strength at failure averaged over four samples (mean ± SD). (**h**) Ultimate tensile strain of 5% *v*/*v* SWPS showing ~35% stretchability before failure.

The electrical response of the SWPS was evaluated by applying incremental pressures uniformly across the sensor surface and recording the resultant capacitance variations (Figure 2a). The initial thickness of the uncompressed SWPS (*d*_0_) was measured prior to loading. Upon the application of external pressure, the porous silk fibroin matrix compresses, reducing both pore dimensions and overall thickness, which in turn modulates the capacitance. Notably, the Young’s modulus of the SWPS is highly tunable through the silk fibroin concentration used during fabrication. As illustrated in Figure 2b, increasing the silk concentration from 1% to 6% *v*/*v* corresponds to a rise in the Young’s modulus from approximately 4 kPa to 35 kPa, indicating progressively stiffer sponges at higher polymer loadings. Sensors cast from 1% to 4% *v*/*v* silk dispersions exhibit high sensitivity in the low-pressure regime (0–900 Pa), but suffer sensitivity loss beyond 900 Pa. In contrast, sensors prepared from 5% and 6% *v*/*v* silk maintain a linear capacitance response throughout 0–2400 Pa, with the 5% device yielding the highest sensitivity. Consequently, a 5% *v*/*v* silk fibroin concentration was selected for all subsequent experiments.

To further elucidate mechanical resilience, SWPS samples were subjected to controlled compressive strains ranging from 10% to 80% (Figure 2c). As the compression ratio increases, the applied pressure required for deformation correspondingly rises. Repeated loading–unloading cycles demonstrate that even under substantial compression, the SWPS preserves both high sensitivity and mechanical recoverability. Quantitatively, Figure 2d plots the change in capacitance (Δ*C*) as a function of applied pressure levels (100, 200, 300, 600, 900, 1200, 1500, 1800, and 2100 Pa). The ΔC values measured at these pressures (3, 5, 7, 14, 18, 22, 27, 33, and 45, respectively) exhibit excellent linearity (R^2^ = 0.99) and yield a calculated sensitivity of 0.0187 Pa^−1^. Importantly, SWPS can detect minute pressures as low as 100 Pa, underscoring its high resolution.

Long-term mechanical and electrical stability were assessed by subjecting the 5 wt% SWPS to cyclic loading at 14 cycles min^−1^ over 1000 s (Figure 2e). Throughout this endurance test of ~230 cycles, the capacitance response remains stable with minimal drift, highlighting the sensor’s robustness for potential long-term monitoring. Further work is needed to validate the device’s long-term durability under continuous operation, specifically beyond 10^4^ loading cycles. In addition to compressive loading, we assessed the tensile performance of the 5 vol% SWPS composite to gauge its stretch resilience. Figure 2f shows a representative tensile stress–strain curve, while Figure 2g,h detail its mechanical limits: the device withstands elongation up to ≈35% strain before failing at a peak stress of ≈44 kPa, demonstrating robust stretchability. This deformation range closely matches the skin strains encountered during joint movement and corroborates our on-body trials (e.g., finger bending), in which the sensor operates reliably under combined tensile and compressive stresses.

### 3.3. Assessment of SWPS Biodegradability and Cytocompatibility

**Figure 3:**
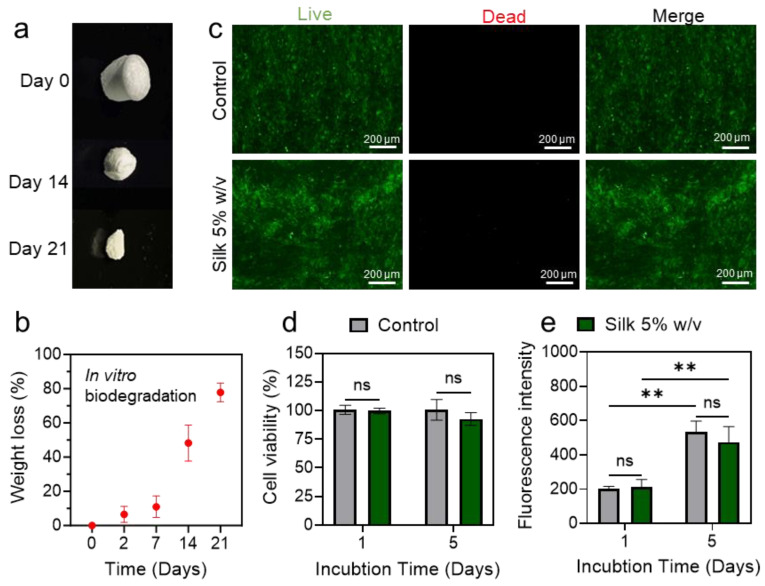
In vitro degradation and biocompatibility of the SWPS. (**a**) Photographic sequence of silk sponge degradation in PBS containing protease over 7, 14, and 21 days. (**b**) Quantitative mass loss of silk sponges during the 21-day enzymatic degradation assay (mean ± SD, *n* = 3). (**c**) Live/dead fluorescence images of HDFs cultured for 5 days on SWPS devices (bottom row) versus tissue culture plastic control (top row). Live cells (calcein AM, green) predominate with minimal dead cells (ethidium homodimer-1, red). Scale bars: 200 µm. (**d**) Percentage viability of HDFs on day 5, comparing AgNW–silk substrates at various concentrations to the control (mean ± SD, *n* = 4). (**e**) PrestoBlue assay quantifying HDF metabolic activity after 1 and 5 days on AgNW–silk sponges at different silk concentrations (mean ± SD, *n* = 4). Statistical significance was determined using one-way ANOVA followed by Tukey’s post hoc test for multiple comparisons. Asterisks exhibit significance levels with *p* < 0.001 (**), and “ns” indicates no statistically significant difference.

Silk fibroin is renowned not only for its biocompatibility and mechanical resilience but also for its rapid and environmentally benign biodegradation, aligning with current priorities in sustainable material design. To evaluate the degradability of the SWPS, a 21-day in vitro assay was performed using phosphate-buffered saline (PBS) containing 2 U mL^−1^ protease XIV at 37 °C. To assess the SWPS’s degradability, we conducted a 21-day in vitro incubation in PBS containing 2 U mL^−1^ protease XIV at 37 °C, a standard accelerated proteolysis condition in the silk biomaterials literature. Although this enzyme concentration exceeds physiological levels, it facilitates an observable mass loss over a 2–3 week period and aligns our results with prior studies of silk-based materials. Enzymatic exposure initiated the gradual breakdown of the silk matrix, with negligible mass loss (<10%) observed after 2 days (Figure 3a,b). Over the full 21-day period, the SWPS lost more than 80% of its initial mass, demonstrating that silk fibroin degrades efficiently under physiological conditions without leaving persistent residues. The inherent biodegradability of our WPS is especially advantageous for transient wearable devices in short-term clinical, athletic, or environmental monitoring. Once their service life concludes, these sensors can be safely discarded, minimizing persistent electronic waste and providing an eco-friendly alternative to conventional, non-degradable materials.

Equally critical for wearable devices is cytocompatibility, particularly in the context of prolonged skin contact and potential leaching of silver nanowires (AgNWs). To assess whether residual silk fibroin or AgNWs elicit cytotoxic responses, human dermal fibroblasts (HDFs) were seeded directly onto porous pure silk sponges or fully assembled SWPS devices and cultured at 37 °C for up to 5 days. Live/dead fluorescence imaging (Figure 3c) revealed predominantly well-spread, viable cells (green) with very few dead cells (red), indicating that neither the silk scaffold nor the integrated AgNW electrodes compromised cell membrane integrity. Quantitative analysis showed that cell viability exceeded 90% by day 5, with no statistically significant differences between control wells (cells on tissue culture plastic) and any of the test samples (Figure 3d). Moreover, metabolic activity, as determined by the PrestoBlue assay, increased steadily from day 1 to day 5 (Figure 3e), confirming robust cell proliferation on both pure silk and SWPS substrates. Importantly, the incorporation of AgNW electrodes did not adversely affect fibroblast viability or growth, thereby validating the cytocompatibility of the complete sensor device. Although our current study centers on HDFs, the silk–AgNW sensor device’s biocompatibility has been comprehensively validated in our previous work [16]. In that study, the subcutaneous implantation in rats elicited only minimal inflammation, low levels of immune cell infiltration, and no evidence of systemic organ toxicity over a four-week period, supporting the sensor’s long-term safety for skin-contact applications.

### 3.4. On-Body Validation of the SWPS

Human study is essential for demonstrating the practical utility of wearable sensors. To evaluate SWPS performance under real-world conditions, we conducted comprehensive on-body tests to assess its ability to transduce complex physiological signals and body movements into electrical outputs.

**Figure 4:**
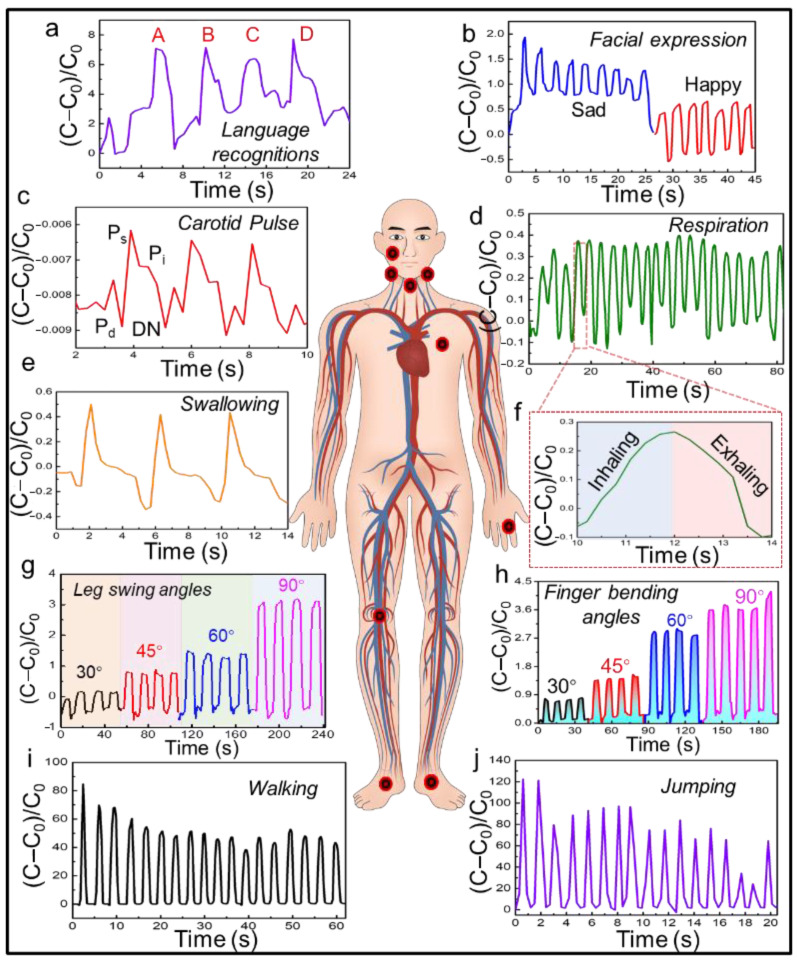
On-body validation of SWPS: real-time capacitance responses during diverse physiological and motion tasks. (**a**) Speech recognition: SWPS positioned near the vocal cords records distinct capacitance peaks corresponding to plosive consonants (“A,” “B,” “C,” and “D”). (**b**) Facial expression monitoring: Sensor affixed to the cheek captures differential capacitance waveforms for happy (top) versus sad (bottom) expressions. (**c**) Carotid pulse detection: SWPS placed over the carotid artery transduces arterial pulsations into reproducible capacitance oscillations, enabling noninvasive heart rate monitoring. (**d**) Continuous respiration tracking: Chest-mounted SWPS registers slow periodic capacitance changes during inhalation (blue) and exhalation (red), as shown in the highlighted inset. (**e**) Swallowing motion: Throat-attached sensor produces transient capacitance spikes corresponding to individual deglutition events. (**f**) Single-breath waveform: A zoomed-in trace from panel (**d**) illustrates the high temporal resolution of inhalation/exhalation phases within one respiratory cycle. (**g**) Leg swing angles: SWPS affixed above the knee records angle-dependent capacitance changes as the leg swings at 30°, 45°, 60°, and 90° flexion. (**h**) Finger flexion: Sensor mounted across a finger joint resolves incremental capacitance peaks when bending at 30°, 45°, 60°, and 90° angles. (**i**) Walking gait analysis: Plantar surface SWPS captures cyclic capacitance patterns during normal walking. (**j**) Jumping dynamics: The same foot-mounted sensor accurately records high-amplitude capacitance spikes corresponding to repeated jumps.

When positioned adjacent to the vocal folds (Figure 4a), the SWPS accurately captured the vibrational signatures associated with distinct phonemes. Sharp, transient electrical peaks corresponded to plosive consonants (e.g., “B,” “D”), whereas smoother, lower-amplitude waveforms were recorded during more sonorous sounds (e.g., “C”). This capability highlights the sensor’s potential for applications in speech recognition and voice-driven human–machine interfaces.

Facial expression monitoring was demonstrated by affixing SWPS devices to regions of facial musculature (Figure 4b). The sensor discriminated against electrical patterns corresponding to expressions of joy and sadness, thereby detecting subtle muscle deformations. Such high-fidelity mapping of emotion-related facial movements could inform research in affective computing, behavioral economics, and real-time emotion recognition. For cardiovascular assessment, the SWPS was placed over the carotid artery (Figure 4c) to record pulse waveforms. The sensor’s high sensitivity enabled real-time acquisition of the carotid pulse amplitude, rhythm, and waveform morphology, suggesting utility in the noninvasive monitoring of heart rate variability, arrhythmia detection, and early cardiovascular diagnostics.

Respiratory dynamics were monitored by mounting the sensor on the chest wall (Figure 4d,f). Subtle thoracic expansions and contractions during inhalation and exhalation produced distinct capacitance–time profiles. Continuous tracking of respiratory rate and pattern can inform pulmonary function monitoring, sleep disorder diagnosis, and the management of obstructive lung diseases. Placement of the SWPS near the larynx (Figure 4e) facilitated real-time detection of swallowing events. Each deglutition induced a transient capacitive spike, providing precise timing and coordination data. This noninvasive approach offers promise for dysphagia assessment and rehabilitation, enabling the quantification of swallow kinematics without requiring cumbersome instrumentation.

The sensor’s flexibility and sensitivity also extend to musculoskeletal applications. When adhered across joints such as the knee and finger (Figure 4g,h), the SWPS exhibited distinct, angle-dependent capacitance shifts across bending angles from 30° to 90°, demonstrating real-time graded deformation sensing and robust reliability under multiaxial strain. The linear relationship between joint flexion and electrical output underscores the device’s potential in gait analysis, physical therapy, and sports science, where the accurate quantification of joint range of motion is critical for performance optimization and injury prevention.

**Figure 5:**
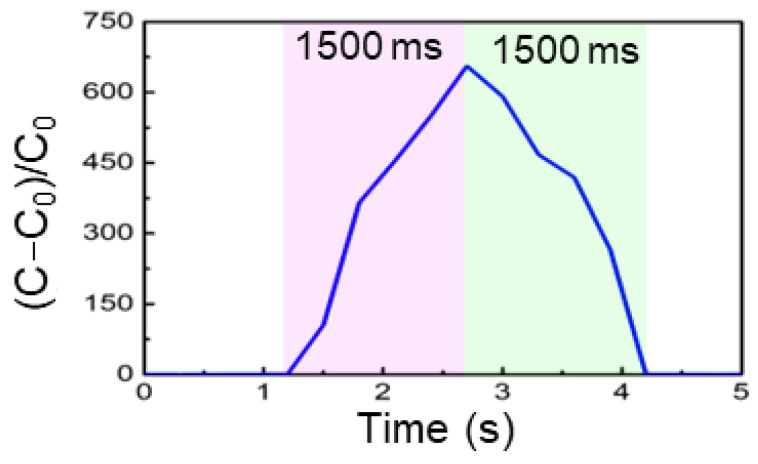
Normalized capacitance change (*C*−*C*_0_)/*C*_0_ of the SWPS under a step load, showing both the rise (pink shaded) and fall (green shaded) phases, each requiring approximately 1500 ms, indicating a total response and recovery time of ~1.5 s.

Finally, SWPS devices placed on the plantar surface (Figure 4i,j) captured dynamic pressure profiles during locomotion. Walking and jumping generated reproducible electrical signals that corresponded to distinct phases of the foot–ground interaction. Figure 5 shows the normalized capacitance change in the SWPS under a step load, with both the rise and recovery phases each taking about 1500 ms. This capability paves the way for advanced gait analysis, rehabilitation monitoring, and athletic performance evaluation, leveraging the sensor’s high resolution and mechanical compliance to map large-scale human movement with precision.

Although comprehensive environmental stress tests were beyond this study’s scope, the sensor maintained stable capacitance outputs during on-body trials despite minor fluctuations in skin temperature and perspiration. Moreover, our previous work [16] demonstrated that the AgNW–silk device preserves its electrical integrity through repeated wet–dry cycles, highlighting its resilience to humidity and temperature changes. Future studies will systematically quantify performance under controlled environmental conditions.

### 3.5. Extreme Load Endurance Testing of the SWPS

**Figure 6:**
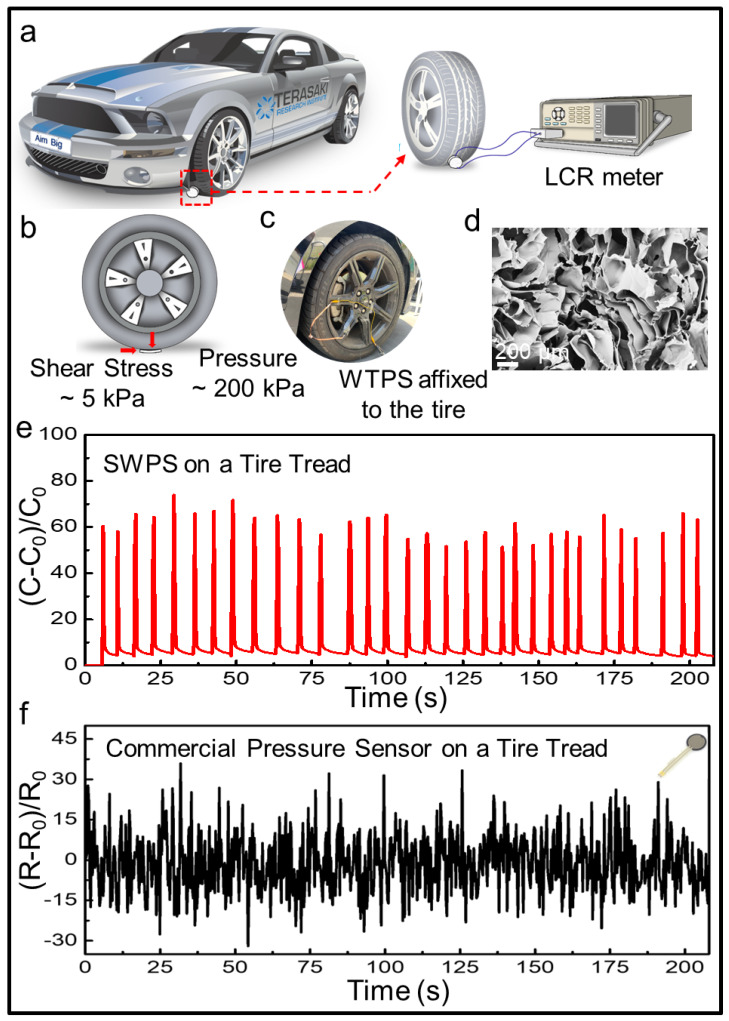
Extreme condition evaluation of SWPS under vehicular loading. (**a**) Schematic representation of the tire rolling test: a SWPS device is affixed to the left front tire of a vehicle and connected to an LCR meter for real-time signal acquisition. (**b**) Illustration of contact stresses: during wheel passage, the sensor experiences approximately 200 kPa of compressive pressure and 5 kPa of shear stress. (**c**) Photograph of a SWPS mounted on the tire tread. (**d**) SEM image of the silk sponge microstructure post-test, showing minimal morphological alteration and confirming the sensor’s structural integrity after repeated high-pressure cycles. (**e**,**f**) Comparative signal output during a 200 s drive at 5 mph: the SWPS (top, red trace) produces sharp, consistent capacitance spikes with each tire rotation, whereas a commercial resistive sensor (bottom, black trace) exhibits noisy, irregular resistance changes under identical conditions.

Pressure testing under extreme loading conditions is essential to evaluate the SWPS’s ultimate robustness and demonstrate its applicability in demanding environments. To this end, we mounted SWPS devices onto the tread surface of an automobile tire and recorded real-time capacitance changes during vehicular passage. As depicted in Figure 6a,c, a SWPS was affixed to the left front tire, with electrical leads connected to an LCR meter for the continuous monitoring of capacitance. When the tire rolled over the sensor, the SWPS experienced both normal pressure and tangential shear forces (Figure 6b). These compressive and shear forces were applied concurrently at varying orientations to replicate the complex, multidirectional deformation profiles encountered in real-world environmental and wearable scenarios. The shear stress can be expressed as follows:

(1) $$\tau =\frac{F}{A},$$

where τ is the shear stress; F is the force applied; and A is the cross-sectional area of material with area parallel to the applied force vector. When the test car is running, the sensor experiences an approximate pressure of 200 kPa and a shear force of about 5 kPa, which is a great challenge for a pressure sensor. To compare the performance of the SWPS under extreme conditions with a commercial resistive pressure sensor, a FlexiForce™ A401 Sensor was chosen and tested. The electrical signals generated by the change in capacitance from both our sensor and commercial sensor were recorded during 200 s driving on the road (Figure 6d). The silk and AgNWs-based sensor was capable of providing real-time feedback with high consistency and accuracy with each rolling motion of the tire. Every time the wheel passed over the SWPS, an obvious and huge capacitance change could be detected, and the amplitude of each signal is basically the same. However, during long-term stress testing, the same as the test on our sensors, commercial sensors were not able to accurately measure each wheel crush, and the resistance signal had a lot of noise. On the contrary, our sensor’s performance remained stable throughout the entirety of the test, proving its resilience and robustness in high-pressure conditions. Figure 6e shows the SEM image of the SWPS after extreme pressure testing. The microstructure of the sensor’s silk sponge showed minimal alteration, reaffirming the sensor’s robustness and capacity to maintain structural integrity even under severe conditions. This robust performance underscores the sensor’s suitability for applications that demand endurance and reliability in the face of extreme pressures.

## 4. Conclusions

In this work, we report on the design of a robust wearable pressure sensor based on silk fibroin/AgNW-sandwiched architecture for medical monitoring. The device demonstrates exceptional accuracy and stability across a wide pressure spectrum, from subtle carotid artery pulsations to dynamic forces generated during walking and jumping. The comparison of the maximum sensitivity versus maximum range with other silk-based sensor devices has been summarized in Table 1. When affixed to the skin, the SWPS delivers precise, real-time feedback on physiological pressures, enabling the early detection of cardiovascular and musculoskeletal anomalies and facilitating personalized therapeutic interventions. Moreover, its inherent biodegradability minimizes the environmental impact commonly associated with disposable wearable electronics. Under extreme loading, such as vehicular tire compression, SWPS outperforms a commercial resistive sensor in both signal fidelity and mechanical resilience. Taken together, these findings establish SWPS as a versatile platform for next-generation, eco-friendly wearable sensors in healthcare applications.

## Figures and Tables

**Table 1:** Comparison of maximum sensitivity versus maximum range with other published data.

Ref.	Material Composition	Sensitivity	Detection Range	Biocompatibility	Extreme Condition Stability
[31]	SEBS/CNT gradient fiber mat	0.068 kPa^−1^ (0–53 kPa); 0.013 kPa^−1^ (53–660 kPa)	0–660 kPa	N/A	Stable after 30,000 cycles, 20 washes
[32]	Silk fibroin diaphragm	12.3 nm/kPa	N/A	Biocompatible	N/A
[33]	P-silk/RG with tunable micropillars	N/A	0.5–200 g	N/A	Waterproof, 8000 cycles
[34]	AgNW electrodes on cotton	N/A	N/A	N/A	N/A
[35]	Single-ply knitted yarn	0.72 kPa^−1^	0.255–35.65 kPa	N/A	Withstands folding/stretching
Ours	SWPS (Silk-AgNW)	18.68 kPa^−1^	0–2.4 kPa (linear)	>90% cell viability; >80% mass loss in 21 days	Tolerates 200 kPa + 5 kPa shear

## Data Availability

Data are contained within the article.

## References

[1] Luo Y., Abidian M.R., Ahn J.-H., Akinwande D., Andrews A.M., Antonietti M., Bao Z., Berggren M., Berkey C.A., Bettinger C.J. (2023). Technology Roadmap for Flexible Sensors. ACS Nano.

[2] Zhu Y., Li J., Kim J., Li S., Zhao Y., Bahari J., Eliahoo P., Li G., Kawakita S., Haghniaz R. (2023). Skin-interfaced electronics: A promising and intelligent paradigm for personalized healthcare. Biomaterials.

[3] Lee Y., Tian X., Park J., Nam D.H., Wu Z., Choi H., Kim J., Park D.-W., Zhou K., Lee S.W. (2025). Rapidly self-healing electronic skin for machine learning–assisted physiological and movement evaluation. Sci. Adv..

[4] Zhang Y., Yang J., Hou X., Li G., Wang L., Bai N., Cai M., Zhao L., Wang Y., Zhang J. (2022). Highly stable flexible pressure sensors with a quasi-homogeneous composition and interlinked interfaces. Nat. Commun..

[5] Han S., Kim J., Won S.M., Ma Y., Kang D., Xie Z., Lee K.-T., Chung H.U., Banks A., Min S. (2018). Battery-free, wireless sensors for full-body pressure and temperature mapping. Sci. Transl. Med..

[6] Park J., Seo B., Jeong Y., Park I. (2024). A Review of Recent Advancements in Sensor-Integrated Medical Tools. Adv. Sci..

[7] Liu X., Zhao P., Wu X., Zhao Y., Zhou F., Luo Y., Jia X., Zhong W., Xing M., Lyu G. (2025). Negative Pressure Smart Patch to Sense and Heal the Wound. Adv. Sci..

[8] Li L., Zheng J., Chen J., Luo Z., Su Y., Tang W., Gao X., Li Y., Cao C., Liu Q. (2020). Flexible Pressure Sensors for Biomedical Applications: From Ex Vivo to In Vivo. Adv. Mater. Interfaces.

[9] Zhu Y., Hartel M.C., Yu N., Garrido P.R., Kim S., Lee J., Bandaru P., Guan S., Lin H., Emaminejad S. (2022). Epidermis-Inspired Wearable Piezoresistive Pressure Sensors Using Reduced Graphene Oxide Self-Wrapped Copper Nanowire Networks. Small Methods.

[10] Xu Y., Sun B., Ling Y., Fei Q., Chen Z., Li X., Guo P., Jeon N., Goswami S., Liao Y. (2020). Multiscale porous elastomer substrates for multifunctional on-skin electronics with passive-cooling capabilities. Proc. Natl. Acad. Sci. USA.

[11] Zhang B., Li J., Zhou J., Chow L., Zhao G., Huang Y., Ma Z., Zhang Q., Yang Y., Yiu C.K. (2024). A three-dimensional liquid diode for soft, integrated permeable electronics. Nature.

[12] Vepari C., Kaplan D.L. (2007). Silk as a biomaterial. Prog. Polym. Sci..

[13] Sahoo J.K., Hasturk O., Falcucci T., Kaplan D.L. (2023). Silk chemistry and biomedical material designs. Nat. Rev. Chem..

[14] Li C., Guo C., Fitzpatrick V., Ibrahim A., Zwierstra M.J., Hanna P., Lechtig A., Nazarian A., Lin S.J., Kaplan D.L. (2020). Design of biodegradable, implantable devices towards clinical translation. Nat. Rev. Mater..

[15] Mirbakht S.S., Golparvar A., Umar M., Kuzubasoglu B.A., Irani F.S., Yapici M.K. (2025). Highly Self-Adhesive and Biodegradable Silk Bioelectronics for All-In-One Imperceptible Long-Term Electrophysiological Biosignals Monitoring. Adv. Sci..

[16] Haghniaz R., Gangrade A., Montazerian H., Zarei F., Ermis M., Li Z., Du Y., Khosravi S., de Barros N.R., Mandal K. (2023). An All-In-One Transient Theranostic Platform for Intelligent Management of Hemorrhage. Adv. Sci..

[17] Wang C., Xia K., Zhang Y., Kaplan D.L. (2019). Silk-Based Advanced Materials for Soft Electronics. Acc. Chem. Res..

[18] Choi W., Heo D., Kim T., Jung S., Choi M., Heo J., Kwon J.-S., Kim B.-S., Lee W., Koh W.-G. (2022). Stress Dissipation Encoded Silk Fibroin Electrode for the Athlete-Beneficial Silk Bioelectronics. Adv. Sci..

[19] Song Y., Hu C., Wang Z., Wang L. (2024). Silk-based wearable devices for health monitoring and medical treatment. iScience.

[20] John D.A., Parameswaran C., Sandhu S., Dahiya R. (2023). Silk Nanofibers-Based Soft and Degradable Capacitive Pressure Sensor Arrays. IEEE Sens. Lett..

[21] Ding S., Jin X., Guo J., Kou B., Chai M., Dou S., Jin G., Zhang H., Zhao X., Ma J. (2025). A biomimetic asymmetric structured intelligent wound dressing with dual-modality humidity-pressure sensing for non-invasive and real-time wound healing monitoring. Adv. Fiber Mater..

[22] Xing T., He A., Huang Z., Luo Y., Zhang Y., Wang M., Shi Z., Ke G., Bai J., Zhao S. (2023). Silk-based flexible electronics and smart wearable Textiles: Progress and beyond. Chem. Eng. J..

[23] Xing C., Luo M., Sheng Q., Zhu Z., Yu D., Huang J., He D., Zhang M., Fan W., Chen D. (2024). Silk Fabric Functionalized by Nanosilver Enabling the Wearable Sensing for Biomechanics and Biomolecules. ACS Appl. Mater. Interfaces.

[24] Lv Q., Li Q., Cao P., Wei C., Li Y., Wang Z., Wang L. (2025). Designing Silk Biomaterials toward Better Future Healthcare: The Development and Application of Silk-Based Implantable Electronic Devices in Clinical Diagnosis and Therapy. Adv. Mater..

[25] Cho D., Li R., Jeong H., Li S., Wu C., Tzavelis A., Yoo S., Kwak S.S., Huang Y., Rogers J.A. (2021). Bitter Flavored, Soft Composites for Wearables Designed to Reduce Risks of Choking in Infants. Adv. Mater..

[26] Kim J., Yoo S., Liu C., Kwak S.S., Walter J.R., Xu S., Rogers J.A. (2023). Skin-interfaced wireless biosensors for perinatal and paediatric health. Nat. Rev. Bioeng..

[27] Liu C., Kim J.-T., Yang D.S., Cho D., Yoo S., Madhvapathy S.R., Jeong H., Yang T., Luan H., Avila R. (2023). Multifunctional Materials Strategies for Enhanced Safety of Wireless, Skin-Interfaced Bioelectronic Devices. Adv. Funct. Mater..

[28] Wray L.S., Hu X., Gallego J., Georgakoudi I., Omenetto F.G., Schmidt D., Kaplan D.L. (2011). Effect of processing on silk-based biomaterials: Reproducibility and biocompatibility. J. Biomed. Mater. Res. B Appl. Biomater..

[29] Wen D.-L., Sun D.-H., Huang P., Huang W., Su M., Wang Y., Han M.-D., Kim B., Brugger J., Zhang H.-X. (2021). Recent progress in silk fibroin-based flexible electronics. Microsyst. Nanoeng..

[30] Zhu Y., Kim S., Ma X., Byrley P., Yu N., Liu Q., Sun X., Xu D., Peng S., Hartel M.C. (2021). Ultrathin-shell epitaxial Ag@Au core-shell nanowires for high-performance and chemically-stable electronic, optical, and mechanical devices. Nano Res..

[31] Jiang J., Song X., Qi Y., Tao X., Zheng Z., Huang Q. (2025). Skin-inspired, permeable, structure-gradient fiber mats for pressure sensing in rehabilitation assistance. Adv. Fiber Mater..

[32] Cheng L., Wang C., Huang Y., Liang H., Guan B.-O. (2016). Silk fibroin diaphragm-based fiber-tip Fabry-Perot pressure sensor. Opt. Express.

[33] Ge D., Mi Q., Gong R., Li S., Qin C., Dong Y., Yu H.-Y., Tam K.C. (2023). Mass-Producible 3D Hair Structure-Editable Silk-Based Electronic Skin for Multiscenario Signal Monitoring and Emergency Alarming System. Adv. Funct. Mater..

[34] Wang S., Zong Q., Yang H., Tan C., Huang Q., Liu X., Zhang G., French P., Ye H. (2023). Rapid Fabrication of High-Performance Flexible Pressure Sensors Using Laser Pyrolysis Direct Writing. ACS Appl. Mater. Interfaces.

[35] Liang Z., Niu M., Xie F., Zhang D., Dai L., Cai X. (2024). A Single-Ply and Knit-Only Textile Sensing Matrix for Mapping Body Surface Pressure. IEEE Sens. J..

